# Effect of bovine respiratory disease on the respiratory microbiome: a meta-analysis

**DOI:** 10.3389/fcimb.2023.1223090

**Published:** 2023-09-08

**Authors:** Samantha Howe, Beth Kegley, Jeremy Powell, Shicheng Chen, Jiangchao Zhao

**Affiliations:** ^1^ Department of Animal Science, Division of Agriculture, University of Arkansas, Fayetteville, AR, United States; ^2^ Medical Laboratory Sciences Program, College of Health and Human Sciences, Northern Illinois University, DeKalb, IL, United States

**Keywords:** meta-analysis, respiratory microbiome, bovine respiratory disease, alpha diversity, differential abundance

## Abstract

**Background:**

Bovine respiratory disease (BRD) is the most devastating disease affecting beef and dairy cattle producers in North America. An emerging area of interest is the respiratory microbiome’s relationship with BRD. However, results regarding the effect of BRD on respiratory microbiome diversity are conflicting.

**Results:**

To examine the effect of BRD on the alpha diversity of the respiratory microbiome, a meta-analysis analyzing the relationship between the standardized mean difference (SMD) of three alpha diversity metrics (Shannon’s Diversity Index (Shannon), Chao1, and Observed features (OTUs, ASVs, species, and reads) and BRD was conducted. Our multi-level model found no difference in Chao1 and Observed features SMDs between calves with BRD and controls. The Shannon SMD was significantly greater in controls compared to that in calves with BRD. Furthermore, we re-analyzed 16S amplicon sequencing data from four previously published datasets to investigate BRD’s effect on individual taxa abundances. Additionally, based on Bray Curtis and Jaccard distances, health status, sampling location, and dataset were all significant sources of variation. Using a consensus approach based on RandomForest, DESeq2, and ANCOM-BC2, we identified three differentially abundant amplicon sequence variants (ASVs) within the nasal cavity, ASV5_*Mycoplasma*, ASV19_*Corynebacterium*, and ASV37_*Ruminococcaceae.* However, no ASVs were differentially abundant in the other sampling locations. Moreover, based on SECOM analysis, ASV37_*Ruminococcaceae* had a negative relationship with ASV1_*Mycoplasma_hyorhinis*, ASV5_*Mycoplasma*, and ASV4_*Mannheimia*. ASV19_*Corynebacterium* had negative relationships with ASV1_*Mycoplasma_hyorhinis*, ASV4_*Mannheimia*, ASV54_*Mycoplasma*, ASV7_*Mycoplasma*, and ASV8_*Pasteurella*.

**Conclusions:**

Our results confirm a relationship between bovine respiratory disease and respiratory microbiome diversity and composition, which provide additional insight into microbial community dynamics during BRD development. Furthermore, as sampling location and sample processing (dataset) can also affect results, consideration should be taken when comparing results across studies.

## 1 Introduction

Bovine respiratory disease (BRD), also referred to as bovine bronchopneumonia, is the most devastating disease affecting North American cattle producers (Taylor et al., 2010). BRD is the leading cause of death in pre-weaned dairy calves (Dubrovsky et al., 2020) and is one of the leading causes of disease affecting feedlot cattle, specifically in the first 50 days post feedlot arrival (Timsit et al., 2016). It accounts for 70-80% of total feedlot morbidity and 40-50% of total feedlot mortality (Edwards, 2010). The USDA APHIS Feedlot study estimated that BRD costs, on average, $23.60/case (USDA-APHIS, 2013). The costs associated with BRD can be attributed to the cost of treatment and decreased carcass quality grade. In the early 2000s, BRD was estimated to cost approximately $800-900 million annually (Brooks et al., 2011), and more recently, it has been estimated to be between $1-3 billion annually in the United States (Cozens et al., 2019).

BRD is considered a multifactorial disease complex with multiple causative agents, the most common being a bacterial infection, typically with *Mannheimia haemolytica, Pasteurella multocida, Histophilus somni*, or *Mycoplasma bovis.* However, the commonly isolated bacterial “pathogens” are often found in the upper respiratory tract (URT) of healthy cattle. Nevertheless, historically most research has focused on these opportunistic pathogens. Recently the role of the respiratory microbiome in BRD has become a major research area of interest. Major differences exist in the URT and lower respiratory tract (LRT) microbiomes of beef and dairy cattle with and without BRD (Lima et al., 2016; Gaeta et al., 2017; Johnston et al., 2017; Zeineldin et al., 2017; Timsit et al., 2018; Klima et al., 2019; McMullen et al., 2019; McMullen et al., 2020; Zeineldin et al., 2020; Raabis et al., 2021; Centeno-Martinez et al., 2022). However, results regarding alpha diversity (intra-sample diversity) and differentially abundant taxa are inconclusive.

In human medicine, it is well-accepted that a loss of microbial diversity in the gastrointestinal tract leads to many diseases (Mosca et al., 2016). Additionally, in cystic fibrosis patients, reduced respiratory microbial diversity has been correlated with reduced respiratory function (van der Gast et al., 2011; Fodor et al., 2012; Zhao et al., 2012). Moreover, decreased alpha diversity is linked to COVID-19 infection and severity (Xu et al., 2021). Decreased richness (Holman et al., 2015; Timsit et al., 2018; McMullen et al., 2019) and decreased Shannon Diversity Index (Timsit et al., 2018) have been observed in the URT of calves with BRD compared to healthy calves. However, several studies have found no significant difference or pattern of change in alpha diversity metrics between BRD and healthy calves (Zeineldin et al., 2017; McMullen et al., 2019; McMullen et al., 2020). Furthermore, it has been observed that calves that developed BRD had decreased richness at arrival compared to those that remained healthy (Holman et al., 2015). However, this has also been disputed, as others observed no difference (Zeineldin et al., 2017). Many studies report slightly different results for observed sequence variants, including observed operational taxonomic units (OTUs) (Holman et al., 2015), species (Zeineldin et al., 2017; McMullen et al., 2019), amplicon sequence variants (ASVs), and the number of reads (Lima et al., 2016), which can make comparisons among studies difficult. Furthermore, previous results have varied regarding differentially abundant and significantly enriched bacterial taxa as well (Lima et al., 2016; Zeineldin et al., 2017; McMullen et al., 2020; Zeineldin et al., 2020; Raabis et al., 2021; Centeno-Martinez et al., 2022; Li et al., 2022).

More and more studies show that meta-analysis is a very powerful tool to evaluate a scientific question when current research is heterogenous, if there are conflicting results, or if there is a lack of consensus regarding a certain scientific question (Siddaway et al., 2019; Tawfik et al., 2019). As both culture-dependent and -independent microbiome studies attempt to describe microbial ecology (Gray and Head, 2008), formal meta-analyses can likely be used to examine microbiome-associated metrics and remove the “noise” that may contribute to conflicting results due to hiding the underlying “biological pattern” (Duvallet et al., 2017; Nikolova et al., 2021). For example, Nikolova et al. (2021) performed a meta-analysis to analyze the effect of gut microbiome alpha diversity associated with numerous psychiatric conditions (Nikolova et al., 2021). Moreover, Avalos-Fernandez et al. (2022) conducted a meta-analysis to determine the effect of respiratory microbiome alpha diversity’s relationship with chronic lung disease (Avalos-Fernandez et al., 2022). However, analyzing reported metrics for relative abundance presents some issues, such as differences in bioinformatic analyses pipelines, classification databases, and differential abundance method, as all of these can affect taxonomic classification and relative and differential abundance results (Edgar, 2018; López-García et al., 2018; Prodan et al., 2020; Nearing et al., 2022). Therefore, compiling and re-analyzing sequences may be a preferred method to examine individual taxa abundances across studies. As a result, a “formal” meta-analysis was conducted to examine the development of BRD on commonly analyzed alpha diversity metrics, including Shannon Diversity Index, Chao1, and variations of observed sequence variants (OTUs, species, ASVs, reads), hereon referred to as “observed features.” Additionally, four datasets [(Nicola et al., 2017) (Nicola), (Johnston et al., 2017) (Johnston), (Centeno-Martinez et al., 2022) (Centeno-Martinez), and PRJNA532923 (PRJNA) (Arkansas, U.o, 2019)] of publicly available sequences were re-analyzed to examine the effect of BRD on individual bacterial abundances.

## 2 Materials and methods

### 2.1 Literature search, data extraction, and effect size calculation

**Figure 1:**
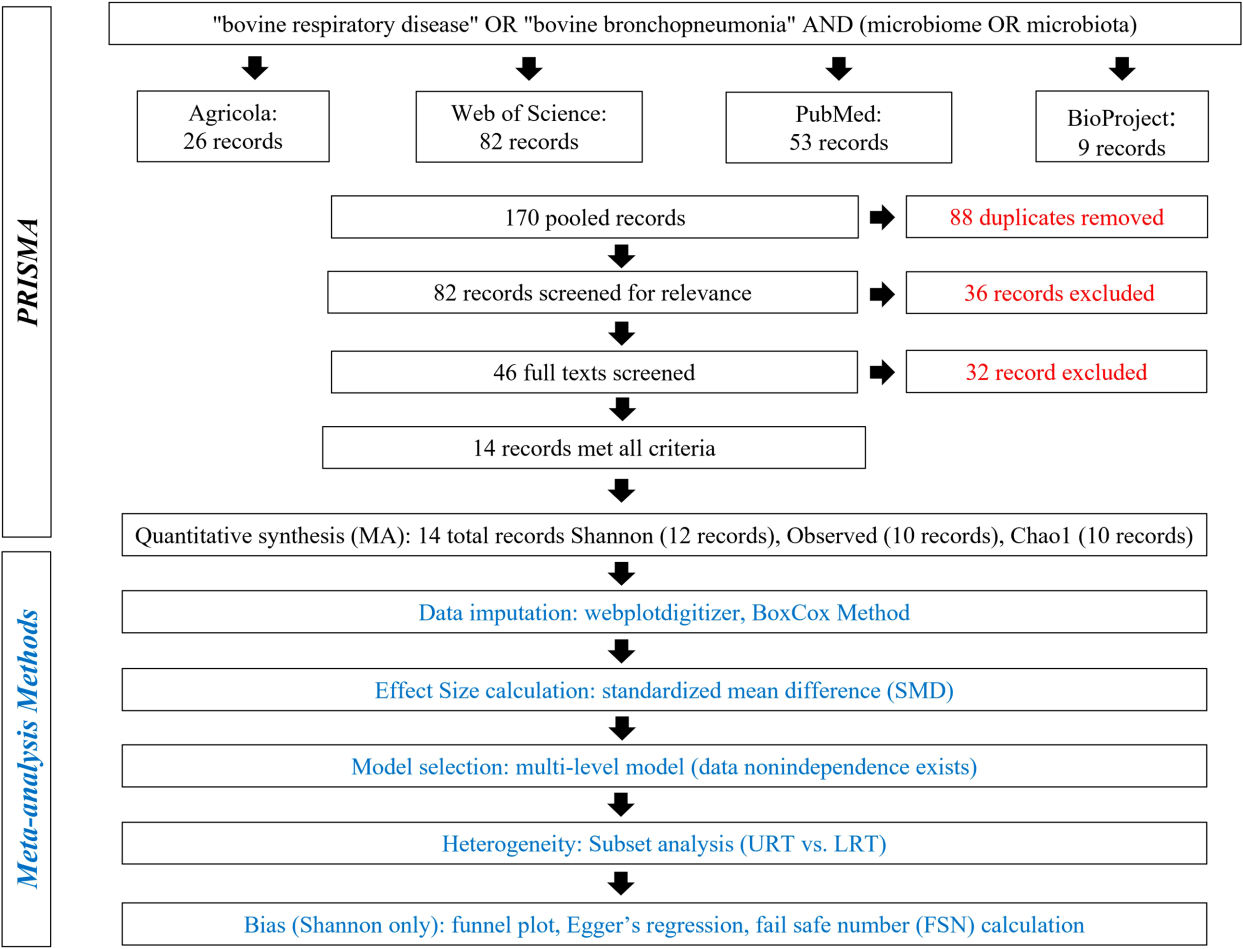
Preferred Reporting Items for Systematic Reviews and Meta-Analyses (PRISMA) for alpha diversity analysis.

A detailed literature search was conducted using Preferred Reporting Items for Systematic Reviews and Meta-analyses (PRISMA) methods on May 5, 2022, and again on November 21, 2022 (**Figure 1**) (Moher et al., 2009). The search terms “bovine respiratory disease” OR “bovine bronchopneumonia” AND “microbiome OR microbiota” were used to search the following databases: Agricola, NCBI PubMed, Web of Science, and NCBI BioProject. Databases were searched to acquire relevant literature and deposited but not published sequences. A total of 170 records were pooled, and 88 duplicates were removed. Then, 82 records were screened for relevance, and 36 were excluded for being not of interest (i.e., were review papers, not in cattle, not microbiome, etc.). The remaining 46 records were screened in more detail and were required to meet the following criteria: compare 16S rDNA sequences from healthy and BRD cattle and report one of the three alpha diversity metrics or contain publicly available data. Finally, 32 records were excluded for not meeting the criteria, and 14 were included in the meta-analysis (**Figure 1**).

Available data ]i.e., alpha diversity metrics’ (Shannon Index, Chao1, and Observed features) mean, standard deviation, and the number of samples in each group (BRD, control)] was extracted from each study. If the mean and standard deviation values were not readily presented in the paper, Webplotdigitizer was used to extract the data from published figures (Rohatgi, 2021). If the only available figures did not provide the mean and standard deviation (i.e., boxplot), these values were imputed using the following website https://smcgrath.shinyapps.io/estmeansd/ using S2 and the Box-Cox method (McGrath et al., 2020; McGrath et al., 2022) as it does not assume normality as demonstrated previously (Avalos-Fernandez et al., 2022). For graphics with error bars, the average standard error of the mean (SEM) or standard deviation was used, and the SEM was computed to standard deviation by multiplying the SEM by the square root of the sample size. Due to variable alpha diversity metrics reported and lack of available data, all metrics (Shannon, Chao1, and Observed features) could not be extracted from all studies. Therefore, studies without the metric of interest were excluded from the effect size calculation for that metric. Two records had sequencing data but did not report alpha diversity metrics. Therefore, these sequences were analyzed using QIIME2 (as described in section 3.3 below), and the Shannon’s Diversity Index, Chao1, and Observed ASVs were calculated.

A database containing relevant metadata and data was constructed (**Supplementary Table 1**). The SMD of both metrics was calculated for each record using the escalc function, where 1 (n1i (sample size), m1i (mean), sd1i (standard deviation) referred to healthy calves, and 2 (n2i, m2i, and sd2i) referred to BRD calves, in the R package metafor (v3.8-1) in R (v4.2.1) (Viechtbauer, 2010; Team, R.C, 2022).

### 2.2 Meta-analytical model, subset analysis, and publication bias

A multi-level meta-analytical model was run to determine the overall effect of BRD on Shannon SMD, Observed features SMD, and Chao1 SMD and to account for potential non-independence and heterogeneity introduced by calculating multiple effect sizes for specific records, as some studies reported metrics of interest for differing sample locations (upper vs. lower respiratory tract) or time points (feedlot entry vs. diagnosis, see **Supplementary Table 1**). This was accomplished using the rma.mv function in the metafor package and by setting “random = ~1|paper_num/count”, in which paper_num is the study number and count is the entry number (**Supplementary Table 1**), and the model was fitted using restricted maximum likelihood (REML).

**Figure 2:**
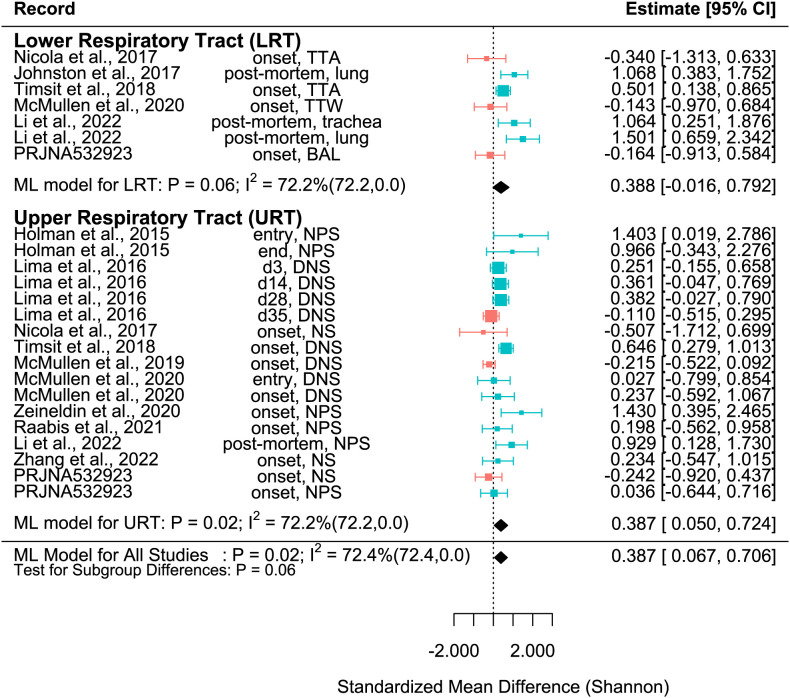
SMD of Shannon Diversity Index in Healthy and BRD Calves. Data located right (blue) or left (pink) of the dotted line indicates that healthy or BRD calves have higher SMD, respectively. Datapoint size corresponds to precision. Bars depict 95% confidence interval. I^2^ indicates total heterogeneity. Values in parentheses indicate between- and within-group heterogeneity. Middle column indicates sampling collection time and sample type [transtracheal aspiration (TTA), transtracheal wash (TTW), bronchoalveolar lavage (BAL), nasopharyngeal swab (NPS), deep nasal swab (DNS), and nasal swab (NS)].

**Figure 3:**
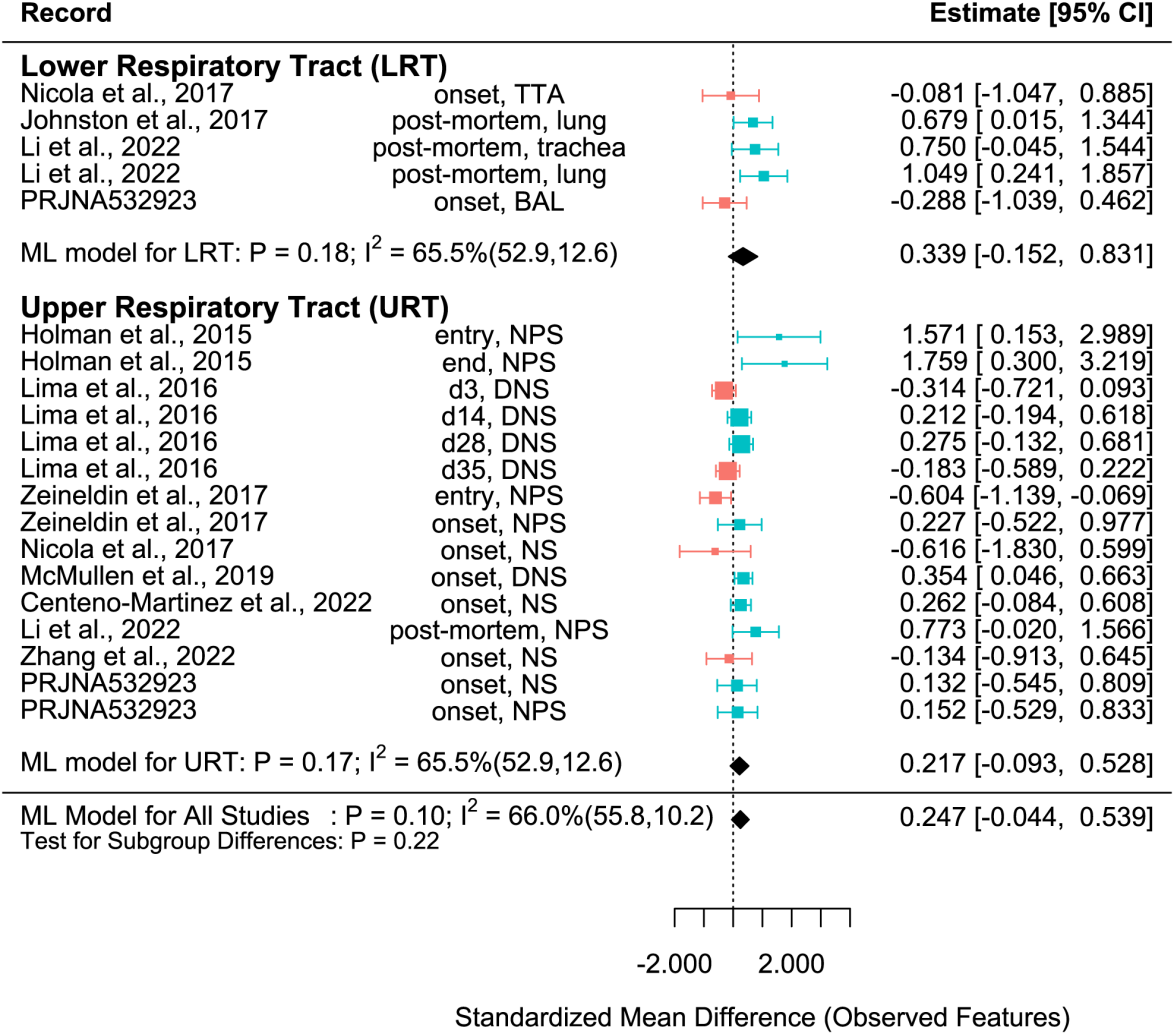
SMD of Observed features in Healthy and BRD Calves. Data located right (blue) or left (pink) of the dotted line indicates that healthy or BRD calves have higher SMD, respectively. Datapoint size corresponds to precision. Bars depict 95% confidence interval. I^2^ indicates total heterogeneity. Values in parentheses indicate between- and within-group heterogeneity. Middle column indicates sampling collection time and sample type [transtracheal aspiration (TTA), transtracheal wash (TTW), bronchoalveolar lavage (BAL), nasopharyngeal swab (NPS), deep nasal swab (DNS), and nasal swab (NS)].

Subgroup analysis was then conducted to determine if sampling location (URT vs. LRT) was a source of residual heterogeneity. Therefore, the previous multi-level model was altered by setting “mods = ~factor(location)-1”. Forest plots of the Shannon, Observed features, and Chao1 SMDs were then created using the forest function in the metafor package (**Figures 1**–**3**).

To assess publication bias for the Shannon SMD, a random effects model was run and used to generate a funnel plot, which was created using the funnel function in the metafor package. Additionally, Egger’s regression test was performed using the regtest function in the metafor package in R. The failsafe number was calculated using the fsn function in the metafor package in R.

### 2.3 Sequencing analysis

The search terms “bovine respiratory disease” OR “bovine bronchopneumonia” AND “microbiome OR microbiota” were used to search NCBI BioProject. To be selected, datasets had to be comprised of 16S sequencing data from the bovine respiratory microbiome from BRD calves and controls, have available metadata indicating which samples were BRD and controls, and be at least overlapping the V4 region. Four datasets met these requirements and were downloaded from the SRA database using prefetch and fasterqdump from the SRA toolkit and were analyzed by QIIME2 (version 2022.8) as previously described (Wang et al., 2019; Wang et al., 2021; Wang et al., 2022). Briefly, data from each dataset were individually imported into QIIME2 (Bolyen et al., 2019). Demultiplexed paired-end V3V4 sequences were trimmed to the V4 region using cutadapt (Martin, 2011) with the forward primer (515F: GTGCCAGCMGCCGCGGTAA) and reverse primer (806R: GGACTACHVGGGTWTCTAAT). The forward and reverse adapters used were the reverse complements of the reverse and forward primer, respectively (forward adapter: ATTAGAWACCCBDGTAGTCC; reverse adapter TTACCGCGGCKGCTGGCAC), and untrimmed reads were discarded. Then for each dataset, forward and reverse reads were joined, reads were filtered, and Deblur was used to further trim and denoise sequences (Amir et al., 2017). Identical filtering and Deblur parameters were used for each dataset. The feature table and representative sequences for each dataset were then merged, and data were rarefied to 1108 reads. Shannon diversity index, Chao1, and Observed ASVs were calculated for alpha diversity analysis (see sections 3.1 and 3.2), and Bray-Curtis and Jaccard distances were calculated using QIIME2 and visualized using PCoA plots created with R (v.4.2.1). ASVs were then classified against the Greengenes database (version 13_8_99), and both relative abundance and count ASV tables were created. Then, Bray Curtis and Jaccard distances were calculated using the distance function in the phyloseq R package (v1.40), and sources of variation were assessed using the anosim function from the vegan package (v2.6-4) (McMurdie and Holmes, 2013).

### 2.4 Differential abundance and taxa interactions

To further investigate the role of BRD on the respiratory microbiome, differential abundance tests were conducted to determine control- and BRD-associated ASVs for each sample type [nasal swabs (NS), nasopharyngeal swabs (NPS), bronchoalveolar lavage (BAL), and trans-tracheal aspiration (TTA)]. Only one of the datasets (Centeno-Martinez) included negative control samples. One ASV (ASV6_*Pseudoalteromonas*) was highly abundant in all negative control samples. This ASV was excluded from NS differential abundance analysis since the Centeno-Martinez dataset was comprised of only NS samples. A consensus approach was used to select differentially abundant taxa for each health status in each sampling location, as recommended by Nearing et al. (2022) (Nearing et al., 2022). To be classified as a control- or BRD- associated taxa, ASVs must have been in the top 25 RandomForest predictors and selected as differentially abundant using both DESeq2 and Analysis of Compositions of Microbiomes with Bias Correction 2 (ANCOM-BC2) for the respective health status. Briefly, RandomForest (v4.7-1.1) was performed on the first 500 ASVs from the rarefied relative abundance table; for each location, a differing number of variables were tried at each branch (mtry) to attempt to optimize results (Breiman, 2001). For DESeq2 and ANCOM-BC2 analysis, the top 1500 ASVs in the rarefied count ASV table were converted into a phyloseq object (McMurdie and Holmes, 2013). For DESeq2 analysis, the phyloseq object was converted to a DESeq2 object, size factors were estimated with the argument type = “poscounts”, and taxa with less than 5 reads in 3 samples were filtered out. Then, DESEq2 (v1.36) with the fittype = “local”, was used to analyze differentially abundant taxa at the ASV level. ASVs were considered differentially abundant using DESeq2 if Padj < 0.05 (Love et al., 2014). For ANCOM-BC2 (v1.6.4) analysis, ASVs with no variances were removed. The following arguments were set as follows, prv_cut = 0.1, p_adj_method = “hochberg”, struc_zero = TRUE, neg_lb = FALSE. ASVs were considered differentially abundant using ANCOM-BC2 if Q < 0.05 (Lin and Peddada, 2020; Lin et al., 2022).

To further examine bacterial relationships at the species level, Sparse Estimation of Correlations among Microbiomes (SECOM) was used to determine both linear and non-linear relationships. The full rarefied count ASV table (excluding the ASV highly abundant in negative controls) was converted into a phyloseq object. Monotonic/linear relationships were quantified using the Spearman correlation coefficient. The secom_linear function in the ANCOMBC package (v2.0.2) was used with the following arguments: pseudo = 0, prv_cut = 0.1, lib_cut = 0, corr_cut = 0.5, wins_quant = c(0.05, 0.95), method = “spearman”, soft = FALSE, n_cv = 10, thresh_hard = 0, thresh_len = 100, max_p = 0.05, n_cl = 2. In order to be further analyzed, taxa had to co-occur in at least ten samples and have P < 0.05. Non-linear relationships were quantified using the distance correlation coefficient. The secom_dist function in the ANCOMBC package was used with the following arguments: pseudo = 0, prv_cut = 0.1, lib_cut = 0, corr_cut = 0.5, wins_quant = c(0.05, 0.95), R = 100, max_p = 0.05, n_cl = 10, thresh_hard = 0 (Lin et al., 2022).

## 3 Results

The literature search resulted in 14 records, including published studies and unpublished deposited sequences. The final database consisted of 27 entries. Information for each entry, such as study sample size, calf age, sample date, and individual study effect sizes can be found in **Supplementary Table 1**. The effect size calculated for both metrics was the standardized mean difference (SMD), also referred to as Hedges g. This method compares the differences between two means, is standardized, and bias-corrected because it takes the sample size into account (Nakagawa et al., 2015). As both metrics were not available for all records, a total of 24, 20, and 18 effect sizes were calculated from 12, 10, and 10 records for Shannon SMD, Observed features SMD, and Chao1 SMD, respectively (**Figure 1**; **Supplementary Table 1**).

### 3.1 Healthy calves have increased Shannon SMD

**Figure 4:**
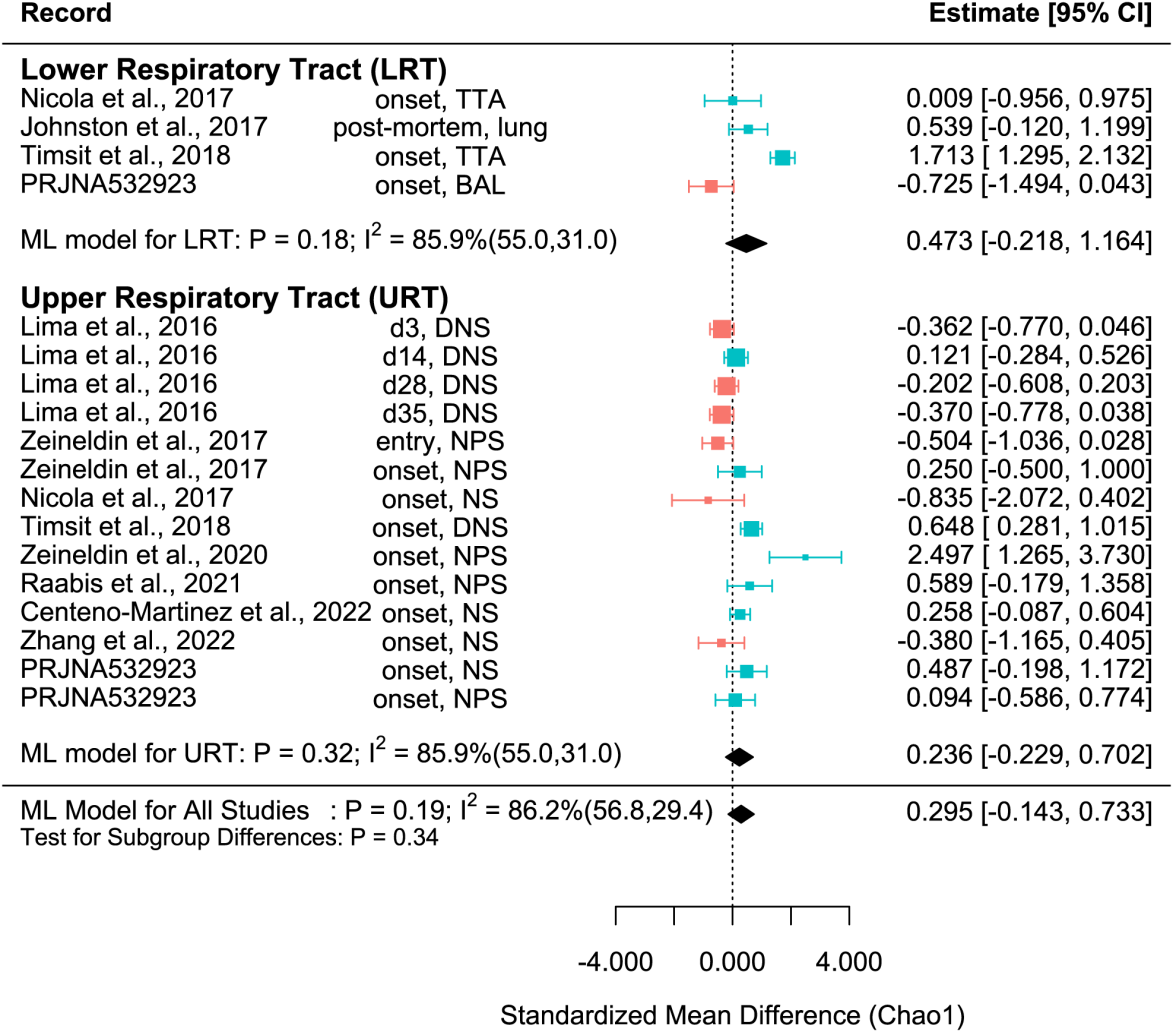
SMD of Chao1 in Healthy and BRD Calves. Data located right (blue) or left (pink) of the dotted line indicates that healthy or BRD calves have higher SMD, respectively. Datapoint size corresponds to precision. Bars depict 95% confidence interval. I^2^ indicates total heterogeneity. Values in parentheses indicate between- and within-group heterogeneity. Middle column indicates sampling collection time and sample type [transtracheal aspiration (TTA), transtracheal wash (TTW), bronchoalveolar lavage (BAL), nasopharyngeal swab (NPS), deep nasal swab (DNS), and nasal swab (NS)].

The SMD varied considerably for each record for all metrics. Among them, many records of Shannon SMD were highly positive, showing that the SMD was greater in healthy calves than in BRD calves. This observation demonstrated that the healthy calves had greater microbial diversity. However, there were still many records with negative SMDs, which signified that the SMD was higher in BRD calves than in healthy calves (**Figure 2**). The same trends were shown in Observed features SMD and Chao1 SMD (**Figures 3**, **4**).

**Figure 5:**
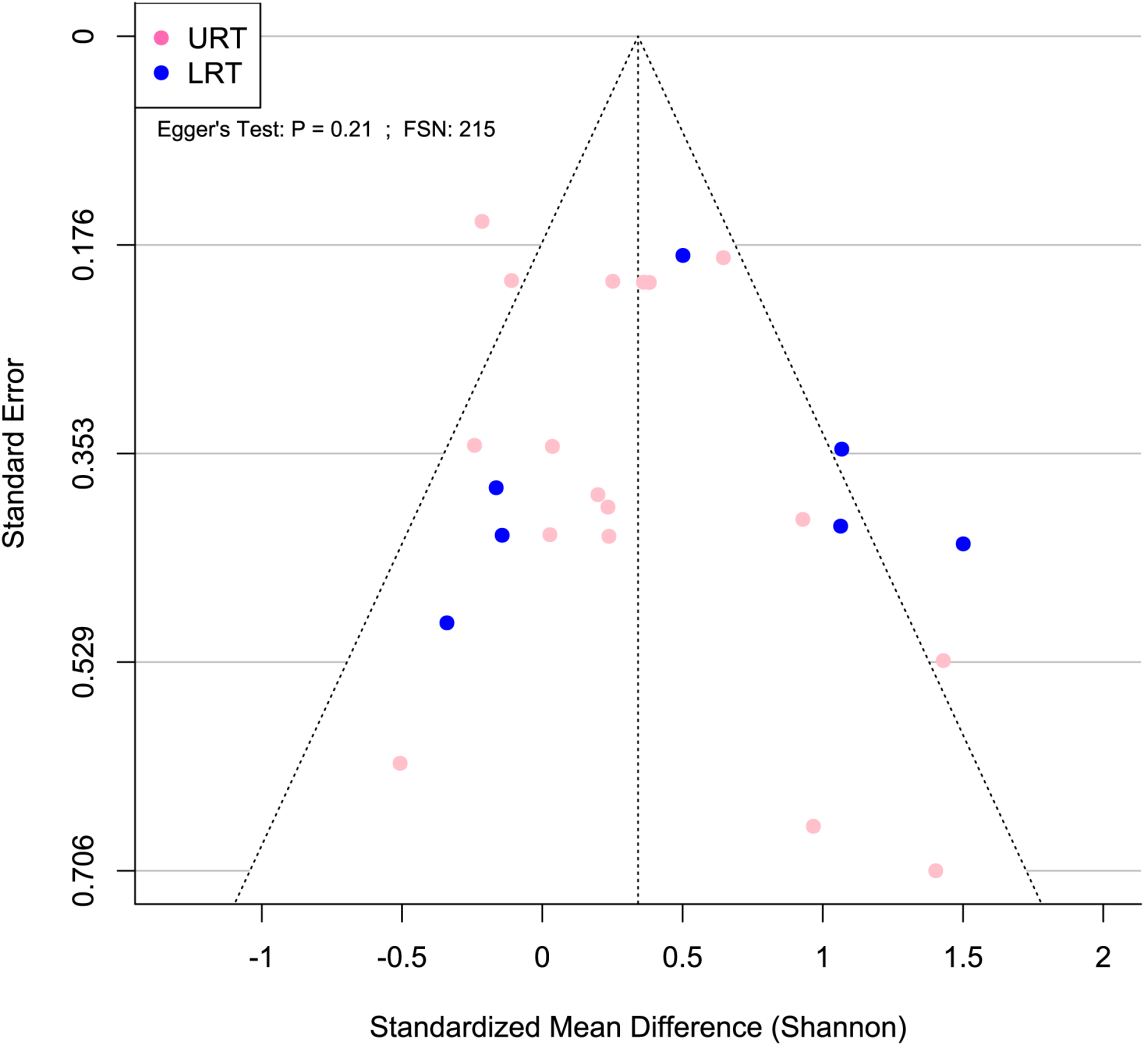
Publication bias was not detected for Shannon SMD. Asymmetrical funnel plot indicates lack of publication bias. Blue and pink circles indicate effect sizes from the lower and upper respiratory tract, respectively. Egger’s test P > 0.05 and FSN > 130 indicate lack of publication bias.

A meta-analytical model determined BRD’s overall effect on the diversity and richness of the respiratory microbiome. Considering the non-independence and heterogeneity introduced by calculating multiple effect sizes from one study, the multi-level meta-analytical model was selected. In this combined analysis, regardless of location, the Shannon SMD was significantly higher in healthy calves than that in calves with BRD (SMD: 0.387, P < 0.05, 95% CI: 0.067 – 0.706). Among them, high heterogeneity was observed (I^2^ = 72.4%) (**Figure 2**). However, there were no significant differences between healthy and BRD calves for Observed features SMD (SMD: 0.247, P > 0.05, 95% CI: -0.044 – 0.539) and only moderate heterogeneity was observed (I^2^ = 66.0%) (**Figure 3**). Similar results were observed for Chao1 SMD (SMD: 0.295, P > 0.10, 95% CI: -0.143 – 0.733) and high heterogeneity was observed (I^2^ = 86.2%) (**Figure 4**). Shannon SMD was next analyzed for publication bias because it was the only significant metric observed. Our result showed that the funnel plot was not asymmetrical and publication bias did not exist based on Egger’s regression test (P > 0.1). The fail-safe number (FSN) was also calculated, indicating the number of insignificant studies needed to decrease the observed significance to the target significance. The FSN for the Shannon SMD model was 215, showing that it would take 215 insignificant results to decrease the observed significance (P < 0.0001) to the target significance (P = 0.05) (**Figure 5**). Additionally, the FSN value was greater than 5k + 10 (where k is the number of effect sizes calculated) (Robert, 1979). Therefore, it was concluded that the Shannon SMD lacked publication bias.

### 3.2 Sampling location (URT vs. LRT) could not explain residual heterogeneity

It is well known that the structure and composition of the upper respiratory tract (URT) and lower respiratory tract (LRT) microbiomes differ (Timsit et al., 2018). In this study, subset analysis was conducted to determine if the sampling sites affected the SMD. In the URT, the Shannon SMD was significantly higher in healthy calves than that in BRD calves (SMD: 0.387, P < 0.05, 95% CI: 0.05 – 0.724). Instead, in the LRT, healthy calves tended to have a higher Shannon SMD compared to BRD calves (SMD: 0.388, P = 0.06, 95% CI: -0.016 – 0.792). Additionally, high heterogeneity still existed after subgroup analysis (I^2^ = 72.2%), indicating that the URT vs. LRT did not explain the heterogeneity observed (**Figure 2**).

For the Observed features SMD, no differences were observed between healthy and BRD calves in the URT (SMD: 0.217, P > 0.1, 95% CI: -0.093 - 0.528) or LRT (SMD: 0.339, P > 0.1, 95% CI: -0.152 – 0.831), and moderate heterogeneity was still observed (I^2^ = 65.5%) (**Figure 3**). Similarly, for the Chao1 SMD, no differences were observed between healthy and BRD calves in the URT (SMD: 0.236, P > 0.1, 95% CI: -0.229 – 0.702) or LRT (SMD: 0.473, P > 0.1, 95% CI: -0.218 – 1.164), and high heterogeneity was still observed (I^2^ = 85.9%) (**Figure 4**).

### 3.3 Sampling location and dataset are major sources of microbiome variation

Four datasets [(Nicola et al., 2017) (Nicola), (Johnston et al., 2017) (Johnston),(Centeno-Martinez et al., 2022) (Centeno-Martinez), and PRJNA532923 (PRJNA) (Arkansas, U.o, 2019)] were used for this study because they contained V4 or V3-V4 16S rDNA sequencing data from the respiratory tract of cattle with and without BRD and included available metadata to identify controls or calves with BRD. However, due to the lack of available data for all time points and unclear metadata, we only used sequences from samples (BRD vs. control) taken at BRD diagnosis/onset or post-mortem samples. Thus, no feedlot entry samples were included in this study. The datasets contained different read numbers; therefore, all samples were further rarefied to 1,108 reads to better examine results evenly across samples.

**Figure 6:**
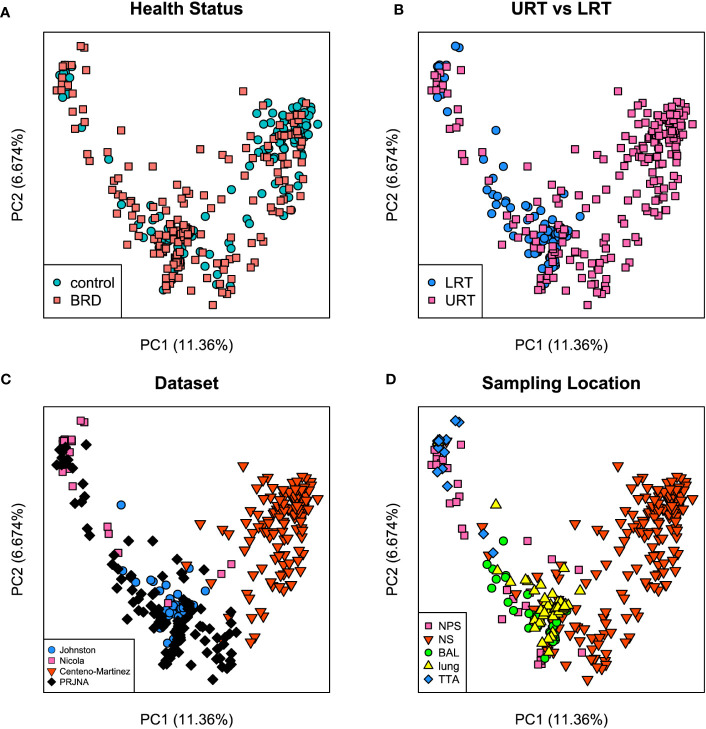
Principal coordinate analysis (PCoA) plots based on Bray-Curtis distances. **(A)** illustrates health status [ANOSIM: R: 0.03, P < 0.05 (**Table 1**)], **(B)** URT vs. LRT [ANOSIM: R: 0.47, P < 0.001 (**Table 1**)], **(C)** dataset [ANOSIM: R: 0.6, P < 0.001 (**Table 1**)], and **(D)** sampling location [ANOSIM: R: 0.64, P < 0.001 (**Table 1**)]. Legends for each plot indicate sample breakdown.

**Figure 7:**
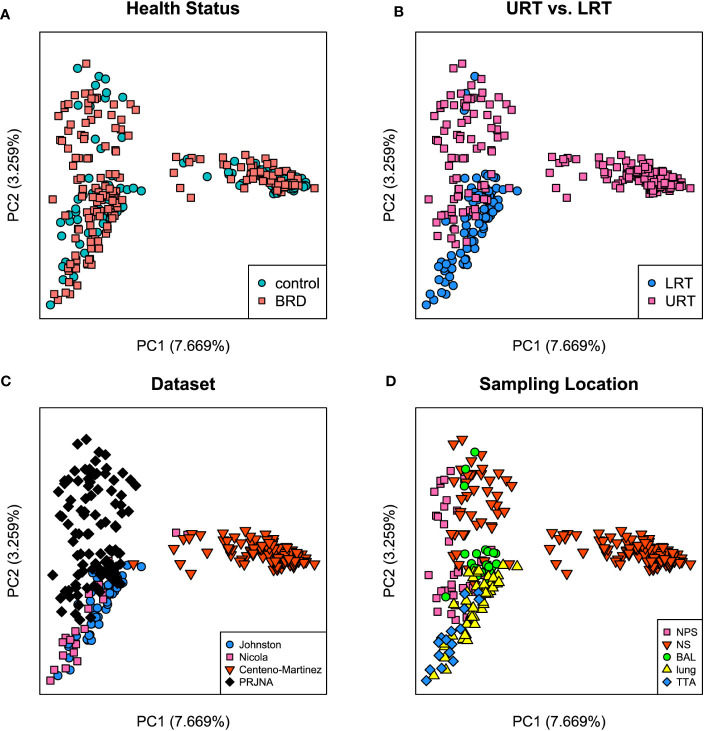
Principal coordinate analysis (PCoA) plots based on Jaccard distances. **(A)** Illustrates health status [ANOSIM: R: 0.047, P < 0.05 (**Table 1**)], **(B)** URT vs. LRT [ANOSIM: R: 0.54, P < 0.001 (**Table 1**)], **(C)** dataset [ANOSIM: R: 0.76, P < 0.001 (**Table 1**)], and **(D)** sampling location [ANOSIM: R: 0.74, P < 0.001 (**Table 1**)]. Legends for each plot indicate sample breakdown.

Both Bray-Curtis and Jaccard distances were calculated and next visualized using PCoA plots. Furthermore, an Analysis of Similarity (ANOSIM) was performed to assess sources of variation. Based on the Bray-Curtis PCoA plot, clustering did not occur due to different health status (*e.g*., BRD vs. controls). However, it occurred when the analysis was done on location within the respiratory tract and dataset (**Figure 6**). This observation was further confirmed by ANOSIM analysis (**Table 1**). Health status, URT vs. LRT, dataset, and sampling location were sources of variation. However, the sampling location (R: 0.6474, P < 0.001) was the most significant source of variation, followed by dataset (R: 0.6032, P < 0.001). Health status contributed the least to variation (R: 0.0399, P < 0.05). Similar results were observed in the analysis of Jaccard distances. Samples did not cluster based on health status and appeared to cluster based on dataset or sampling location (**Figure 7**). Additionally, ANOSIM analysis of Jaccard distances showed that all tested variables were significant sources of variation. However, the main sources were dataset (R: 0.7614, P < 0.001), followed by sampling location (R: 0.7486, P < 0.001); the health status was the least important source of variation (R: 0.0475, P < 0.05) (**Table 1**).

**Table 1:** Analysis of Similarities (ANOSIM) of Bray-Curtis and Jaccard Distances.

	Bray-Curtis R	Jaccard R
Health Status	0.0399^**^	0.04753^***^
URT vs LRT	0.4711^***^	0.5403^***^
Dataset	0.6032^***^	0.7614^***^
Sampling Location	0.6474^***^	0.7486^***^

**P < 0.01.

***P < 0.001.

### 3.4 Control- and BRD- associated ASVs

**Figure 8:**
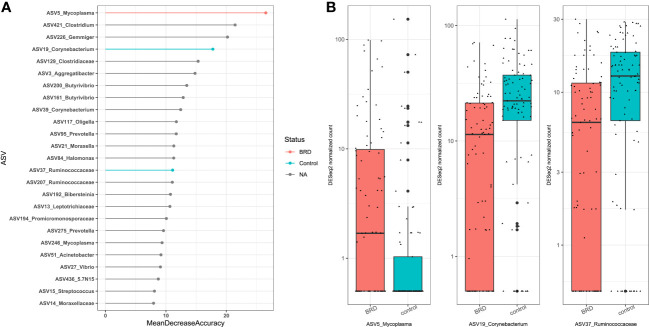
Differentially abundant ASVs within the nasal cavity. ASVs were determined differentially abundant if they were selected as a RandomForest predictors (top 25) and were differentially abundant using both DESeq2 (Padj < 0.05), and ANCOM-BC (Q < 0.05). **(A)** Top 25 RandomForest predictors; pink indicates BRD-associated ASV; blue indicates healthy control-associated ASV. **(B)** Boxplot of DESeq2 counts; Pink indicates abundance in BRD samples; blue indicates abundance in control samples.

Our results showed that sampling location was the largest source of variation based on Bray Curtis distances and the second largest source of variation based on Jaccard distances (**Table 1**); therefore, control- and BRD- associated ASVs were determined based on sampling site, including nasal (NS) (Centeno-Martinez, Nicola, PRJNA), nasopharyngeal (NPS) (PRJNA), lung (Johnston), bronchoalveolar lavage (PRJNA), and trans tracheal aspiration (TTA) (Nicola). ASV6 was removed from NS differential abundance analysis because it was highly abundant in the negative controls of the Centeno-Martinez dataset. A consensus approach was used to determine differentially abundant ASVs, as recommended by Nearing et al. (2022). To be considered differentially abundant, an ASV had to be ranked among the top 25 RandomForest predictors and be selected as differentially abundant using both DESeq2 and ANCOM-BC2. Using these criteria, only one sampling location (NS) contained differentially abundant ASVs. Using DESeq2, 16 ASVs were identified as differentially abundant in the nasal cavity, and 8 of them were RandomForest predictors (**Supplementary Figures 1A, B**). Using ANCOM-BC2, 3 ASVs were identified as differentially abundant in the nasal cavity, which overlapped with those identified by RandomForest and DESeq2. Therefore, the consensus-based approach allowed us to identify 3 differentially abundant ASVs in the nasal cavity. ASV5_*Mycoplasma* was differentially abundant in BRD calves (*DESeq2:* log_2_fold change (lfc): -1.85, p_adj_ < 0.05; *ANCOM-BC2:* W_control_: -4.00, Q < 0.05), and ASV19_*Corynebacterium* (*DESeq2:* lfc: 0.97, p_adj_ < 0.05; *ANCOM-BC2:* W_control_: 4.02, Q < 0.05) and ASV37_*Ruminococcaceae* (*DESeq2:* lfc: 0.82, p_adj_ < 0.05; *ANCOM-BC2:* W_control_: 3.91, Q < 0.05) were differentially abundant in controls (**Figure 8**). Furthermore, the abundance of the three control- or BRD- associated ASVs were also broken down by dataset to ensure that they were present in more than one dataset (**Supplementary Figure 2**).

In NPS samples, 4 ASVs were differentially abundant using DESeq2. Furthermore, two ASVs overlapped with RandomForest predictors. However, no ASVs were identified as differentially abundant using ANCOM-BC2 (**Supplementary Figures 3A, B**). Similar results were observed in the LRT samples. For example, for lung samples, 6 ASVs were differentially abundant when DESeq2 was applied. In this analysis, 1 ASV was found to be differentially abundant using ANCOM-BC2 While no ASVs overlapped with each other. Among TTA samples, only 1 ASV was differentially abundant using DESeq2, while none were differentially abundant using ANCOM-BC2. Moreover, no ASVs were identified as differentially abundant in BAL samples when both DESeq2 and ANCOM-BC2 were used.

### 3.5 Linear and non-linear interactions exist between bacterial taxa in the nasal cavity

To further investigate the possible role(s) of the control- and BRD- associated ASVs, we used SECOM to analyze both linear and non-linear relationships between different taxa (at the ASV level). The nasal cavity was chosen for SECOM analysis because it was the only location with differentially abundant ASVs and contained multiple datasets for comparison. ASV5_*Mycoplasma*, ASV19_*Corynebaterium*, and ASV37_ *Ruminococcaceae* were of interest at the ASV level (**Supplementary Tables 2, 3**). It is noted that there can be non-linear relationships between taxa when no linear relationship exists. However, if a linear relationship exists, a non-linear one should also; if one does not, results should be interpreted carefully. Therefore, only linear relationships with overlapping non-linear relationships were reported. It should also be noted that the non-linear relationship (distance correlation) was only non-zero and does not have a direction, as it only described a general dependency between taxa (Lin et al., 2022). As this was a meta-analysis and combining datasets could introduce lots of “noise,” the primary goal was to identify general dependencies between the selected taxa and any ASVs associated with *Mycoplasma, Mannheimia, Histophilus*, and *Pasteurella*. Therefore, the SECOM distance matrix was primarily used to determine relationships (**Table 2**; **Supplementary Table 2**), and SECOM Spearman2 (p-value filtering) was used to determine the direction of the relationship (**Table 3**; **Supplementary Table 3**). ASV19_*Corynebacterium* had a negative relationship with ASV1_*Mycoplasma_hyorhinis* (distance: 0.38; ρ: -0.41; P < 0.05), ASV4_*Mannheimia* (distance: 0.46; ρ: -0.31; P < 0.05), ASV54_*Mycoplasma* (distance: 0.4; ρ: -0.41; P < 0.05), ASV7_*Mycoplasma* (distance: 0.31; ρ: -0.28, P < 0.05), and ASV8_*Pasteurella* (distance: 0.38; ρ: -0.4; P < 0.05) and a positive relationship with ASV376_*Mycoplasma* (distance: 0.64; ρ: 0.67; P < 0.05). ASV37_*Ruminococcaceae* had a negative relationship with ASV1_*Mycoplasma_hyorhinis* (distance: 0.31; ρ: -0.29; P < 0.05), ASV4_*Mannheimia* (distance: 0.39; ρ: -0.3; P < 0.05), and ASV5_*Mycoplasma* (distance: 0.3; ρ: -0.28; P < 0.05). ASV5_*Mycoplasma* had a positive relationship with ASV1_*Mycoplasma_hyorhinis* (distance: 0.43; ρ: 0.42; P < 0.05), ASV10_*Histophilus_somni* (distance: 0.52; ρ: 0.50; P < 0.05), and ASV346_*Mycoplasma* (distance: 0.88; ρ: 0.87; P < 0.05) (**Tables 2**, **3**). Finally, ASV37_*Ruminococcaceae* had a relationship with ASV7_*Mycoplasma* (distance: 0.22; P < 0.05), but the direction could not be determined (**Table 2**). Furthermore, ASV19_*Corynebacterium* and ASV37_*Ruminococcaceae* had a positive relationship with each other (distance: 0.63; ρ: 0.56; P < 0.05) (**Tables 2**, **3**). These data indicated that, in the nasal cavity, the healthy control-associated ASVs potentially interact with the BRD-associated ASV and other opportunistic pathogens within the nasal cavity and that the BRD opportunistic pathogens were likely interacting with each other.

**Table 2:** Sparse Estimation of Correlations among Microbiomes (SECOM) Distance. Matrix quantifying non-linear relationships between taxa in the bovine nasal cavity.

	ASV1 *Mycoplasma_hyorhinis*	ASV10 *Histophilus_somni*	ASV346 *Mycoplasma*	ASV37 *Ruminococcaceae*	ASV376 *Mycoplasma*	ASV4 *Mannheimia*	ASV5 *Mycoplasma*	ASV54 *Mycoplasma*	ASV7 *Mycoplasma*	ASV8 *Pasteurella*
**ASV19** ***Corynebacterium***	0.379826	0	0	0.626393	0.642521	0.461004	0	0.399021	0.314158	0.377679
**ASV37** ***Ruminococcaceae***	0.307754	0	0	1	0	0.395374	0.299882	0	0.222071	0
**ASV5** ***Mycoplasma***	0.433682	0.52168	0.8803	0.299882	0	0	1	0	0	0

Values are distance correlation coefficients.

Non-zero values indicate general dependency/relationship between taxa (P < 0.05).

Zeroes indicate no dependency/relationships exist between taxa.

**Table 3:** Sparse Estimation of Correlations among Microbiomes (SECOM) Spearman (P value filtering) Matrix quantifying monotonic/linear relationships between taxa in the bovine nasal cavity.

	ASV1 *Mycoplasma_hyorhinis*	ASV10 *Histophilus_somni*	ASV346 *Mycoplasma*	ASV37 *Ruminococcaceae*	ASV376 *Mycoplasma*	ASV4 *Mannheimia*	ASV5 *Mycoplasma*	ASV54 *Mycoplasma*	ASV7 *Mycoplasma*	ASV8 *Pasteurella*
**ASV19** ***Corynebacterium***	-0.411968738	0	0	0.558790593	0.666666667	-0.312895125	0	-0.406967023	-0.284981088	-0.39992
**ASV37** ***Ruminococcaceae***	-0.291410913	0	0	1	0	-0.301406873	-0.284311214	0	0	0
**ASV5** ***Mycoplasma***	0.417471755	0.505609498	0.866666667	-0.284311214	0	0	1	0	0	0

Non-zero values indicate a monotonic/linear relationship between taxa (P < 0.05).

Negative values indicate a negative relationship, and positive values indicate a positive relationship (P < 0.05).

Zero values indicate no monotonic/linear relationships between taxa.

## 4 Discussion

Our study provides the first meta-analysis examining BRD’s effect on the respiratory microbiome alpha diversity. Overall, healthy calves had a greater Shannon diversity index of 0.39 standard deviations, indicating that the respiratory microbiome of healthy calves had increased microbial diversity compared to calves that developed BRD (P < 0.05). These results agreed with the previous observations: healthy calves had increased alpha diversity metrics compared to those with BRD (Holman et al., 2015; Timsit et al., 2018; McMullen et al., 2019). However, no difference was observed for either the Chao1 or Observed features metrics. There could be many reasons for this. First, the Shannon Diversity Index is a measure of evenness and richness, meaning that it considers both bacterial presence/absence and bacterial abundance (Kim et al., 2017). Whereas both Chao1 and Observed features measure bacterial richness, meaning they only take into account bacterial presence/absence (Hughes et al., 2001). Therefore, this might indicate that the abundance of specific bacteria, rather than just their presence, accounted for BRD. However, it is also possible that this difference is not related to disease but rather the effect of different data analytical methods. Chiarello et al. (2022) observed that data analysis pipeline significantly influenced presence/absence indices. Furthermore, richness estimates were also influenced using either ASVs or OTUs. They also noted that this affected both the richness metric values and sample ranking (Chiarello et al., 2022). Therefore, it is possible that the data analysis method had a greater effect on the richness alpha diversity metrics (Chao1, Observed features) than on the Shannon Diversity Index. The effect of data analytical pipeline on alpha diversity SMD could be addressed by re-analyzing publicly available sequencing data. However, many of the included studies did not have publicly available sequencing data and metadata. Future studies should make all sequencing data and metadata publicly available to address this question.

Furthermore, the URT of healthy calves had a greater Shannon diversity index of 0.38 standard deviations than BRD calves (P < 0.05), and no differences were observed in the LRT between BRD and healthy calves. This could indicate that the URT microbiome’s Shannon Diversity Index is more affected by BRD, but it could also be due to the small sample size for the LRT. Although the LRT samples had by far the smallest sample size, the sample size for the entire meta-analysis (total records/effect sizes) is small, indicating a need for additional research into the bovine respiratory microbiome and its role in BRD. In addition, subset analysis based on sampling location did not explain the high heterogeneity observed for Shannon SMD. This indicates that other unexplained factors, such as sampling time, diet, age, and/or management factors, etc., likely also affect the respiratory microbiome, leading to the observed high residual heterogeneity. Future meta-analyses should attempt to explain this heterogeneity if possible. Nevertheless, no publication bias was observed for the Shannon SMD in this study. Therefore, although high residual heterogeneity was observed for Shannon SMD, the lack of publication bias and significant effect size, especially for the URT, clearly demonstrate that the URT microbiome Shannon diversity index is higher in healthy calves than in those with BRD. However, it remains unclear if the decreased diversity in BRD calves is due to disease development or if decreased diversity predisposes the calf to BRD because all sampling time points were analyzed together due to a lack of available data.

To assess the effect of BRD on the respiratory microbiome, publicly available sequences with available metadata were compiled and analyzed as a singular dataset. Previous datasets with available data could not be included due to a lack of available metadata or the use of a non-overlapping 16S hypervariable region. Beta diversity analysis indicated that all tested variables (health status, URT vs. LRT, sampling location, and dataset) were significant sources of variation on both Bray Curtis and Jaccard distances. Interestingly, health status was the smallest source of variation for both beta diversity metrics, whereas either sampling location or dataset were the greatest sources of variation. This indicates that sampling location and other outside influences affect the microbiome more than health status alone. Chai et al. (2022b) performed a meta-analysis on metagenomic sequencing data from the bovine respiratory microbiome. Their results showed that microbial structure and function were significantly affected by both geographical location and sampling niche. Therefore, this indicates that respiratory tract location and environmental factors, such as diet or climate, likely affect the respiratory microbiome more than health status alone, as these differed for each geographic location included in the study (Chai et al., 2022b).

To address these issues, we split samples into individual sampling locations (NS, NPS, BAL, lung tissue, and TTA) to examine control- and BRD-associated ASVs. ASVs were considered differentially abundant using a consensus approach of RandomForest, DESeq2, and ANCOM-BC2. Nearing et al. (2022) showed that differing differential abundance analysis methods yielded differing results and recommended applying a consensus approach to determine robust biological interpretations (Nearing et al., 2022). In the nasal cavity, ASV19_*Corynebacterium* and ASV37_*Ruminococcaceae* were identified as healthy control-associated ASVs, as they were higher in abundance in controls, and ASV5_*Mycoplasma* was identified as the only BRD-associated ASV. While *Mycoplasma* is present in the microbiome of clinically healthy cattle also (Chai et al., 2022a), it is clear that ASV5_*Mycoplasma* is associated with BRD.

To further evaluate the nasal cavity microbiome, Sparse Estimation of Correlations among Microbiomes (SECOM) was used to examine both linear and non-linear relationships between taxa. Pearson and Spearman correlation coefficients have been deemed inappropriate for use with microbiome data; however, SECOM takes into account the sparsity of microbiome data (Lin et al., 2022). Using SECOM at the ASV-level, both ASV37_*Ruminococcaceae* and ASV19_*Corynebacterium* were positively correlated with each other and were negatively correlated with ASV4_*Mannheimia* and many *Mycoplasma* ASVs. Furthermore, ASV19_*Corynebacterium* was negatively correlated with ASV8_*Pasteurella*, and, interestingly, positively correlated with ASV376_*Mycoplasma*. It should be noted that *Mannheimia, Mycoplasma*, and *Pasteurella* are not opportunistic pathogens themselves and that other species exist within these genera as well (Slack, 2010; Suástegui-Urquijo et al., 2015; Parker et al., 2018). *Ruminococcaceae* is a normal and abundant member of the bovine gastrointestinal tract microbiome (Lopes et al., 2019). It has also been observed in the upper respiratory tract as well, and it is thought that its presence is due to contact with feces, manure, or digesta due to rumination (Amat et al., 2019a; Crosby et al., 2022). Additionally, Crosby et al. (2022) observed that *Ruminococcaceae* abundance was decreased in the upper respiratory tract of cattle with BRD and speculated that this might be due to decreased rumination in sick calves (Crosby et al., 2022). Therefore, our observation of ASV37_ *Ruminococcaceae* as a healthy control-associated ASV within the bovine nasal cavity indicates a need for further research into the role of gastrointestinal tract-associated microbes in respiratory health. Amat et al. (2019b) observed that three *Corynebacterium* isolates from the nasopharyngeal tract of healthy cattle were able to inhibit the growth of *M. haemolytica in vitro* (Amat et al., 2019b). One can assume that *Corynebacterium* may repress the growth of BRD pathogens in the animal hosts. It is well established that *Corynebacterium* is part of normal microbiota in the bovine nasal cavity and nasopharyngeal tract regardless of health status (Gaeta et al., 2017; Holman et al., 2017; Nicola et al., 2017; Zeineldin et al., 2017; McDaneld et al., 2018; Timsit et al., 2018; Amat et al., 2019a; Chai et al., 2022a). However, *Corynebacterium* sp. have often been associated with human respiratory disease (Yang et al., 2018) and cause disease in cattle, albeit not in the respiratory tract (Smith et al., 2020; Lücken et al., 2022). Regardless, our results and Amat et al. (2019b) showed that *Corynebacterium* may be involved in maintaining microbiome stability of the bovine URT and preventing BRD opportunistic pathogen invasion and colonization. However, additional research is needed to evaluate the role of *Corynebacterium* within the bovine respiratory tract and the role of gastrointestinal microbes in the upper respiratory tract.

It should be noted that our study has some limitations. First, all data included in this study was based on 16S sequencing data. Future studies utilizing shotgun metagenomics, metabolomics, or quantifying total bacterial load would provide additional insight into the respiratory microbiome’s role in BRD development and progression. Additionally, due to a lack of available data, all time points were analyzed together when analyzing the effect of BRD on different alpha diversity metrics SMD. However, our multi-level model attempted to account for the potential non-independence introduced by calculating multiple effect sizes for some studies. Although, it should be noted that the inclusion criteria for this study were very broad; therefore, only broad conclusions can be drawn. It remains unclear if the decreased Shannon SMD observed in BRD calves is due to disease development or if decreased diversity predisposes the calf to BRD. Future studies should aim to make all data, including sequencing data or calculated alpha diversity metrics (even if the metric is not significant), publicly available to aid in answering this question. Moreover, subset analysis based on sampling location did not explain the high heterogeneity observed for Shannon SMD, indicating that other unexplained factors exist that also affect the respiratory microbiome. Future studies should aim to make all data publicly available and have clear, descriptive metadata, so future meta-analyses can explain the residual heterogeneity observed. Additionally, other factors, such as breed, age, geographical location, climate, and management practices all affect the respiratory microbiome, and we cannot rule out the effects of these factors in our study. Future studies comparing the respiratory microbiome of BRD calves would also need to be standardized in this field, as in the human microbiome project, including sample collection, storage, DNA extraction, the hypervariable region of the bacterial 16S rRNA gene, and analytical pipelines. The definition and diagnosis of BRD, the application of antibiotics, and the sampling time after feedlot arrival should also need to be considered when comparing the bovine respiratory microbiome between studies. Finally, as previously noted, the sample size of our study is small, and many studies could not be included due to either a lack of publicly available data or unclear metadata. Our study would be strengthened by the inclusion of additional data, so future studies should aim to make all sequencing data and descriptive metadata publicly available.

## 5 Conclusion

Our study investigated the effect of BRD on the alpha (intra-sample) diversity of the cattle respiratory microbiome, which fills the knowledge gap between respiratory microbiome alpha diversity and BRD. The multi-level model concluded that healthy calves had an increased Shannon Diversity Index, and no difference was observed for richness measures (Observed or Chao1). Overall, these results indicate that Shannon Index in calves with BRD is lower than in healthy calves. Furthermore, publicly available sequences were combined and re-analyzed for four datasets. ANOSIM, based on Bray-Curtis and Jaccard distances, found that, although significant, health status was the smallest source of variation, and sampling location and dataset were the largest sources of variation, respectively. Additionally, in the nasal cavity, ASV19_*Corynebacterium* and ASV37_*Ruminococcaceae* were healthy control-associated ASVs, and ASV5_*Mycoplasma* was the only BRD-associated ASV. Based on SECOM analysis, ASV19_*Corynebacterium* was negatively associated with ASV4_*Mannheimia*, ASV1_*Mycoplasma_hyorhinis*, ASV54_*Mycoplasma*, ASV7_*Mycoplasma*, and ASV8_*Pasteurella*, and positively correlated with ASV376_*Mycoplasma*, and ASV37_*Ruminococcaceae*, the other healthy control-associated ASV. Taken together, these results indicate that additional research is needed into the role of *Corynebacterium* in the bovine respiratory microbiota and that sampling location and other factors significantly affect microbial structure and need to be considered.

## Data availability statement

Publicly available datasets were analyzed in this study. This data can be found here: All sequencing data used in this study were obtained and were previously available on NCBI Bioproject under the following accessions: PRJNA532923, PRJNA746809, PRJNA383722, PRJNA335827.

## Author contributions

JZ and SH contributed to conception. SH contributed to data collection, analysis, interpretation, and drafted the manuscript. SH, JZ, JP, BK, and SC contributed to critically revised the manuscript. All authors contributed to the article and approved the submitted version.

## Supplementary material

Supplementary Figure 1 ASVs identified as differentially abundant in nasal cavity using only RandomForest and DESeq2. ASVs were selected if they were selected as a RandomForest predictors (top 25) and were differentially abundant using DESeq2 (Padj < 0.05). **(A)** Top 25 RandomForest predictors; pink indicates BRD-associated ASV; blue indicates healthy control-associated ASV. **(B)** Boxplot of DESeq2 counts; Pink indicates abundance in BRD samples; blue indicates abundance in control samples.

Supplementary Figure 2 Differentially abundant ASVs in the nasal cavity broken down by health status and datasets. ASVs were determined differentially abundant if they were selected as a RandomForest predictors (top 25) and were differentially abundant using both DESeq2 (Padj < 0.05), and ANCOM-BC (Q < 0.05). Boxplots formed based on DESeq2 counts. Pink indicates abundance in BRD samples; blue indicates abundance in control samples.

Supplementary Figure 3 ASVs identified as differentially abundant in nasopharyngeal cavity using RandomForest and DESeq2. ASVs were selected if they were selected as a RandomForest predictors (top 25) and were differentially abundant using DESeq2 (Padj < 0.05). **(A)** Top 25 RandomForest predictors; pink indicates BRD-associated ASV; blue indicates healthy control-associated ASV. **(B)** Boxplot of DESeq2 counts; Pink indicates abundance in BRD samples; blue indicates abundance in control samples.
