# Supplementary figures and images for: Effect of bovine respiratory disease on the respiratory microbiome: a meta-analysis
Source: Front Cell Infect Microbiol. 2023 Sep 8;13:1223090. doi: 10.3389/fcimb.2023.1223090 (PMC10516580; doi:10.3389/fcimb.2023.1223090)

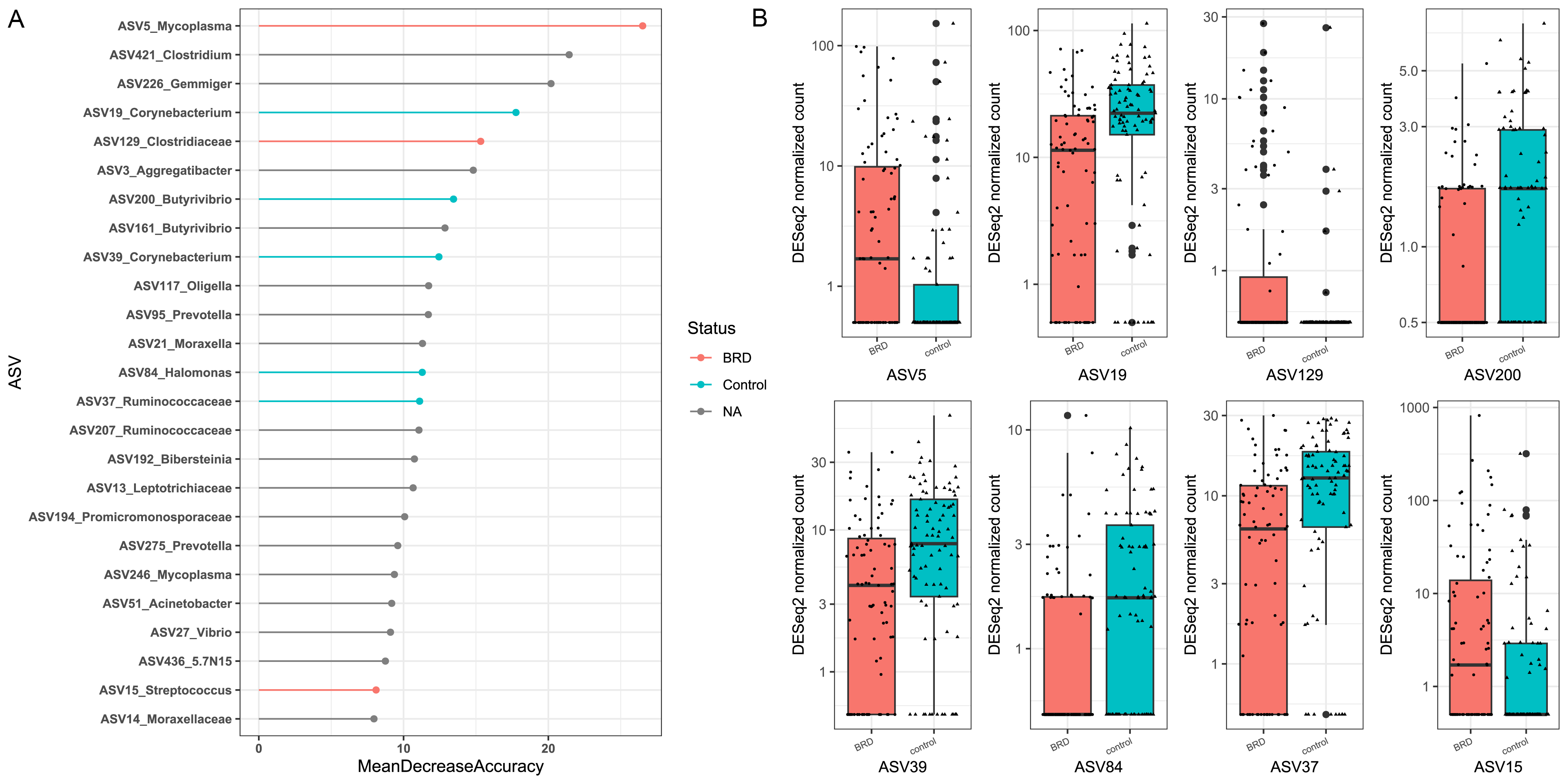

Supplement: Supplementary Figure 1 — ASVs identified as differentially abundant in nasal cavity using only RandomForest and DESeq2. ASVs were selected if they were selected as a RandomForest predictors (top 25) and were differentially abundant using DESeq2 (Padj < 0.05). (A) Top 25 RandomForest predictors; pink indicates BRD-associated ASV; blue indicates healthy control-associated ASV. (B) Boxplot of DESeq2 counts; Pink indicates abundance in BRD samples; blue indicates abundance in control samples. [file Image_1.tif]

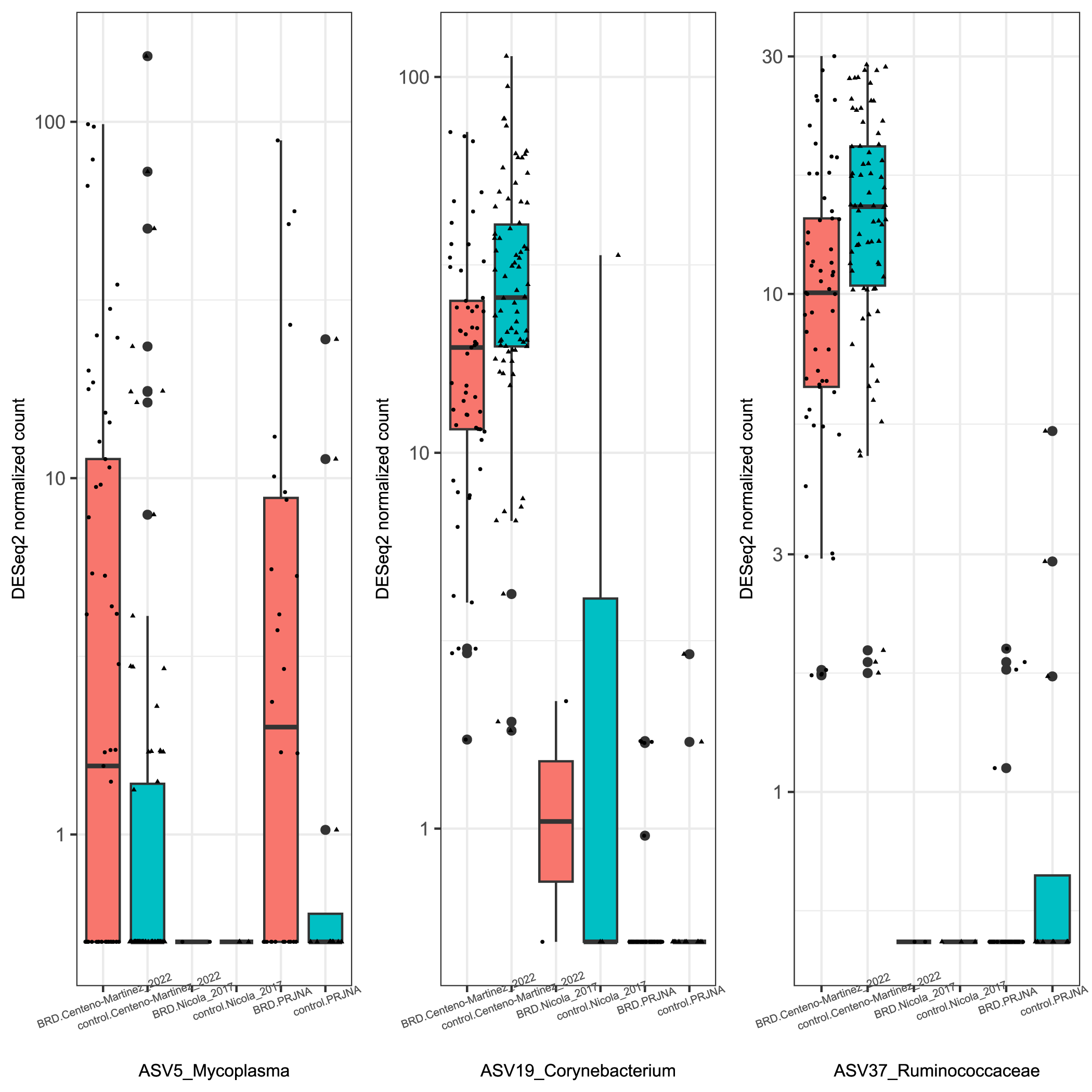

Supplement: Supplementary Figure 2 — Differentially abundant ASVs in the nasal cavity broken down by health status and datasets. ASVs were determined differentially abundant if they were selected as a RandomForest predictors (top 25) and were differentially abundant using both DESeq2 (Padj < 0.05), and ANCOM-BC (Q < 0.05). Boxplots formed based on DESeq2 counts. Pink indicates abundance in BRD samples; blue indicates abundance in control samples. [file Image_2.tif]

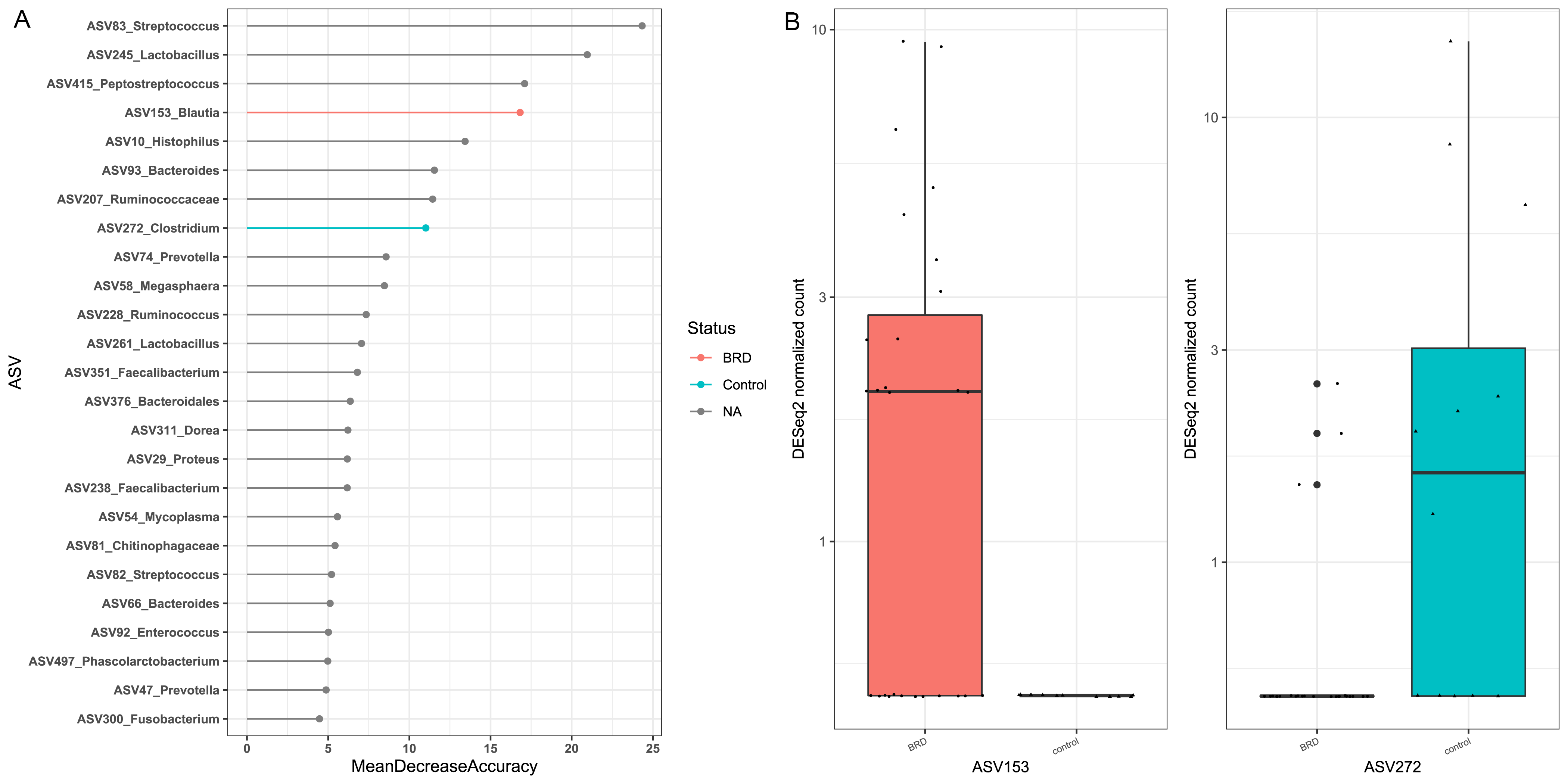

Supplement: Supplementary Figure 3 — ASVs identified as differentially abundant in nasopharyngeal cavity using RandomForest and DESeq2. ASVs were selected if they were selected as a RandomForest predictors (top 25) and were differentially abundant using DESeq2 (Padj < 0.05). (A) Top 25 RandomForest predictors; pink indicates BRD-associated ASV; blue indicates healthy control-associated ASV. (B) Boxplot of DESeq2 counts; Pink indicates abundance in BRD samples; blue indicates abundance in control samples. [file Image_3.tif]
